# Multimodal Sensory-Spatial Integration and Retrieval of Trained Motor Patterns for Body Coordination in Musicians and Dancers

**DOI:** 10.3389/fpsyg.2020.576120

**Published:** 2020-11-17

**Authors:** Aija Marie Ladda, Sarah B. Wallwork, Martin Lotze

**Affiliations:** ^1^Functional Imaging Unit, Diagnostic Radiology and Neuroradiology, University of Greifswald, Greifswald, Germany; ^2^IIMPACT in Health, Allied Health and Human Performance, University of South Australia, Adelaide, SA, Australia

**Keywords:** parietal lobe, musicians, spatial representation, training, sensorimotor, dancers

## Abstract

Dancers and musicians are experts in spatial and temporal processing, which allows them to coordinate movement with music. This high-level processing has been associated with structural and functional adaptation of the brain for high performance sensorimotor integration. For these integration processes, adaptation does not only take place in primary and secondary sensory and motor areas but also in tertiary brain areas, such as the lateral prefrontal cortex (lPFC) and the intraparietal sulcus (IPS), providing vital resources for highly specialized performance. Here, we review evidence for the role of these brain areas in multimodal training protocols and integrate these findings into a new model of sensorimotor processing in complex motor learning.

## Introduction

We are often perplexed by the precision of spatiotemporal coordination of complex movement patterns in athletes, musicians, and dancers. These processes are not only accurate at a single subject level but also function at the group level forming a synchronization of social coordination. Moving in unity as a group is one of the most pleasurable events for social interaction between humans.

While emotional aspects like arousal and motivation certainly play a central role in entraining motor action to rhythmic auditory stimulation (Bowling et al., 2019), we here would like to focus on the role of the lateral prefrontal cortex (lPFC) in storing movement patterns and the intraparietal sulcus (IPS) in generating movement trajectories that can easily be modified and updated online to enable amazing performances of dance and music. In this review, we describe the neurofunctional basis of the brain processes, which might represent highly specialized performance in dancers and musicians. Firstly, we focus on the processing of the dimensions time and space in the brain that enables a coordination of the body and an interaction with the environment. We discuss that these temporal processes vary between sensory domains and that temporal and spatial integration is a complex process performed by adaptive and hierarchically organized neural networks. We then differentiate models that underpin our understanding of spatial and temporal processing in the brain. In the spatial domain, we explain the construct of the “body matrix” theory as a basis for understanding the functional connection between body, space, cognition, and emotion. In the temporal domain, we provide evidence for anticipative processing in the brain that facilitates synchronization, rhythm, or a cohesive sequence of events. This neurobiological strategy also enables fast interactions, as corrections can be rapidly made in anticipation of performance outcomes. We then go on to explain how these temporal anticipative processes might be represented in the brain. In the second section, we provide more specific details surrounding musical and dance performance. In the third section, we integrate the themes from sections one and two and discuss techniques that may facilitate high-level motor learning in performers that allows individuals to further refine and master highly complex and multimodal tasks.

### The Representation of Timing and Space in the Brain

For both musical performance and dance, the exact timing of a movement in (instrumental) space is essential. Timing precision is on a level of tenth of seconds (Keller et al., 2014) and – especially for instrumental interaction, such as in bands, chamber musicians, and orchestras – has to be adapted to other instrumentalists and singers. Using modern techniques of kinematography in chamber orchestras, Timmers et al. (2020) describe how artistic movement interactions are coordinated with a timing within 50 ms. These timing interactions are highly important for the esthetic experience of the audience. Keller (2014) suggested a framework, which subsumes the factors that influence rhythmic interpersonal coordination in the context of ensemble performance. He stated that temporally precise rhythmic interpersonal coordination requires three core cognitive-motor skills: anticipation, attention, and adaptation. It has been documented that professional pianists or violinists would spend more than 7,500 h of accumulated practice time with their instrument before the age of 18, demonstrating significant time and practice dedicated to refining these skills (Ericsson et al., 1993). This training helps to refine sensorimotor behavior as well as the ability to execute it with the upmost precision of timing in the visual and auditory domain (Cicchini et al., 2012).

Consider the true complexity of human behavior – the ability to perceive and interpret auditory and visual stimuli and then react with their own sounds (vocal or *via* instrument) or movements, which are complex in their own right, to be in perfect harmony with other musicians and/or dancers. The correct perception of temporally near acoustic events such as the experience of fusion or distinction of two succeeding events (fusion threshold) or the recognition of order of two events (order threshold) are predominantly processed in the Broca’s area of the left brain hemisphere (for an overview, see Pöppel, 1996). However, space and time are intermingled: the order of timing of auditory events depends on our position in relation to the external sound producer. Even more complex, timing is different between modalities: whereas the auditory cortex has access to inputs from the ear after several milliseconds, visual input has a temporal delay of about 100 ms until incoming light on the retina is represented as a conscious percept in the primary visual cortex (due to biochemical processes in the cones and rods within the retina) as measured by visually evoked potentials (VEPs). For somatosensory input, such as an electrical stimulus to the finger tips, the earliest cortical signals (SEP, somatosensory evoked potentials) have been described to be ~20–30 ms. This differential delay of cortical information presence between different sensory domains challenges the time-object continuity recognition on a perceptive level. For an object out of reach, the different velocities of sound and light are of importance too. There is a learned time-delay within the processing of auditory and visual signals that can account for these differences in velocities, with processing delays and integration of multisensory stimuli taking place in the brain. The 10 m distance from the object to the receiver (i.e., the brain), where the perception of two sensory stimuli becomes “harmonious” for the visual and auditory system is called “Ereignishorizont” – the horizon of temporal similarity of events (Pöppel et al., 1990). Anything closer than 10 m is processed faster in the auditory domain, and further than 10 m is processed faster in the visual domain. That is why table tennis players, who play in a relatively confined area, respond faster to auditory signals. Athletes that play within larger distances – sports such as tennis or football – are faster on visual stimuli. One’s perception of space and time is sculptured by the interaction of the body to signals emitted from objects in the environment. Minimal alterations in spatial settings, however, for instance in an opera house with sound reflection, can make the identification of a sound-emitting object quite demanding (Moore and King, 1999).

The ability to receive, process, and interpret differing transmission rates of light and sound stimuli as a byproduct of the one event in ever-changing conditions highlights the complexity of the central nervous system. It raises the question – is there a delay function, like an oscillator in a computer, producing subunits of temporal synchrony in a 30 Hz frequency towards consciousness (Pöppel, 1996)? Although, even these time frames seem to be quite short when considering the temporal delay of the visual system – especially when object identification processes can take up to half a second. Here, the model suggested by Wolpert and Ghahramani (2000) of continuous state estimation and feedback integration, might be not only relevant for reducing sensorimotor interactions for objects within reach, but also for adjusting ones’ own movements with objects that are more distant. Besides continuous readjustment of the sensorimotor system, the role of an internal motor engram is critical for highly trained movement patterns in instrumentalists, singers, and dancers. An inverse model of the movement generated during training (blueprint of movement, as named by Rijntjes et al. (1999), or a movement engram, as relevant for movement observation) can then be readjusted in a more economic fashion and applied in the manipulation of an external object. This motor pattern engram is likely represented in the bilateral ventral premotor cortex (vPMC; Rijntjes et al., 1999; Pau et al., 2013) and therefore – at least for the dominant left hemisphere – very near to the timing and language areas of the brain. Repetitive training of movement patterns increases their precision, such that anticipated timing can be incredibly accurate, even if the movement is just imagined (Guillot and Collet, 2005). This engram of the movement pattern of one’s own body is constantly re-corrected with the sensory input: the anterior cerebellar hemisphere integrates anticipated and actual sensory input, functioning as a continuous movement pattern correction. These readjustments are likely processed as corticocerebellar parallel loops. Aside from the motor engram, we can here differentiate three parallel sensorimotor processes, which are continuously readjusted: (1) cortico-cortical loops, including primary and secondary sensory areas, primary and secondary motor areas, and tertiary areas, such as ventrolateral premotor cortex/Broca’s area and the parietal cortex; (2) cerebellar feedforward processing and readjustment with actual sensory input; and (3) movement automatization and smoothing of highly trained movement patterns in the basal ganglia.

Very simple cortico-cortical loops include the interconnectedness of the somatosensory cortices with the primary motor cortex. A feedback-loop model was proposed by Hebb (1949), who suggested that the synchrony of motor output with somatosensory input would dictate a neural network loop. In such established networks, synchronous firing of all network elements can therefore be provoked, even if only one of the elements is addressed (e.g., by a stimulus). This is also highly relevant for musical performance: auditory feedback during training activates these tight loops internally, which can be called upon even in the absence of the motor element (Lotze et al., 2003) or the auditory element (D’Ausilio et al., 2006). This is also true for rhythmic performance in dancers: motor areas can become active to the rhythm, even in the absence of movement (for a review, see Levitin et al., 2018). With respect to temporal cortical processes, we might differentiate between motor sequencing (e.g., fast finger tapping predominantly processed in the supplementary area; SMA, supplementary motor area), more complex coordination of movement patterns (dorsal premotor cortex), temporally dictated movement representations (vPMC, Broca’s area), and space (parietal cortex). Further, cerebellar circuits may work as an ongoing parallel control loop, which corrects anticipated movement patterns and internal timing of movement patterns (Braitenberg et al., 1997) with feedback from sensory information. The complexity of these parallel loops can only be interpreted through neural network processing models, additional synaptic modulation, and regional specification of brain areas. It is also important to mention that internal models are highly dependent on brain maturation and infant development. Whereas spinal and brainstem processes are already completely automated during early development, most other levels of movement coordination and sensorimotor interaction are tracked by repetitive training.

**Figure 1:**
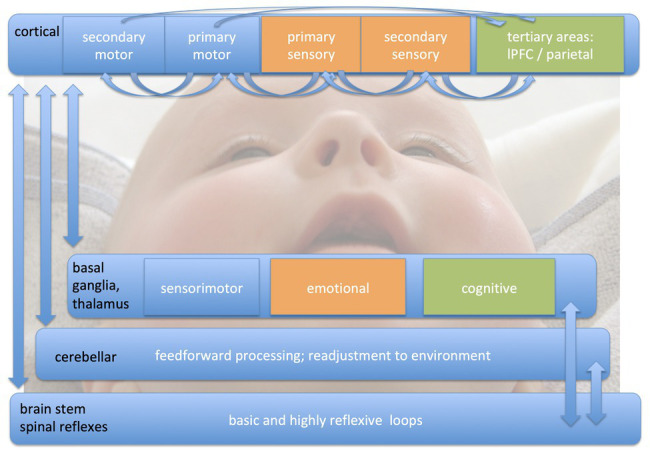
Different loops of sensorimotor processing. There are four different parallel processing loops, which are deferentially shaped by sensorimotor training during different phases of development. We here would like to emphasize the importance of early infancy (as illustrated by the picture of the baby in the background of the figure) in the development and maturation of complex sensorimotor interactions. Whereas brain stem mechanisms and spinal reflexes can be modulated by even a small amount of training, the other three loops (cortical, basal ganglia, and thalamus, and cerebellar) are in a continuous reshaping process. The cortical level is interacting with at least three hierarchy levels from primary to tertiary cortical areas. This occurs for movement execution, timing, bilateral coordination, force level, and sensorimotor integration. The more frequently a movement pattern is executed, the more the basal ganglia are involved in sensorimotor processing. These interactions are not only important for automation, but also are critical in movement modulation. The thalamus integrates all sensory input (except olfactory) and is in a continuous interchanging inhibition/excitation relationship with the cortex. Cerebellar circuits help to integrate feedforward processing with readjustment to the environment.

An overview on what might be developed and experienced in the months before and after birth, when these internal models are processed to an elaborated state which enables training of highly complex movement patterns in the fields overviewed here, is depicted in Figure 1 (oriented to Yang et al., 2018).

Spatial and temporal processing of multisensory stimuli involves highly complex neural networks and loops. The artificial distinction between movement timing and spatial movement processes is brought on by constructs in experimental settings, derived mainly from modular models of movement representation. When thinking about the integration of spatiotemporal information, we should therefore keep in mind that in the context of movement, information about time and space is functionally processed in a somewhat similar way, even if the respective brain areas may be spatially distinct.

### Different Domains of Sensory Integration in Motor Performance

Auditory feedback processing has the most obvious importance for professional musical production. Increased size of auditory perception fields has been reported for professional musicians (Pantev et al., 1998). Somatosensory input is however also important for precise movement guidance in performances on a musical instrument. Increased sensory receptive field sizes have been reported in professional violinists for the left, string-playing hand (Elbert et al., 1995). A tight sensorimotor interaction is trained by repetitive pairing of motor output and received sensory input (e.g., for auditory-motor coupling, see Bangert et al., 2006; Zatorre et al., 2007). Analyses of brain network activity during music listening showed action-based processing in musicians compared to non-musicians (Alluri et al., 2017). In musicians, auditory-motor interactions are facilitated by coupling of the bilateral SMA with the superior temporal gyrus. Differences in the interplay between primary sensorimotor areas and feedforward areas of the anterior cerebellar hemisphere can already be found in amateurs playing either the piano or the trumpet (Gebel et al., 2013). An anticipation of lip movement in trumpet players increases anterior cerebellar feedforward loops in the lip area even if just the finger play on the valves is practiced, without interaction of the lips with the trumpet mouthpiece occurring. These anticipated cerebellar loops are absent when piano players without any experience in playing the trumpet perform the same movements. In singers, the body is the instrument – therefore somatosensory aspects of the areas producing the voice are more in the foreground (Kleber et al., 2007). If the somatosensory larynx area is anesthetized, singing performance is hampered. However, the extent to which performance is hampered is dependent on singing experience, such that experienced singers may compensate for the somatosensory deficit (Kleber et al., 2013). Here, the right anterior insula with the primary somatosensory cortex (S1) is responsible for performance differences in pitch accuracy during singing with laryngeal anesthesia. The right anterior insula was described as a “hub” for experience-dependent modulation of sensorimotor integration of singing performance.

For dancers, moving through space to a specific time-course requires the utmost finesse in motor control and coordination. It is reliant on the ability to precisely control the timing, grading, and magnitude of muscle contractions, as well as perceive small perturbations within the system and react accordingly. During movement, such as in dance, motor commands are sent to the periphery to activate specific muscles, which act in synergy at the joints to form coordinated movement. A copy of the motor command loops back into the central nervous system (i.e., the efference copy) to enable a comparison against the multisensory feedback – comparing the expected outcome to the real-time outcome (see Wolpert and Ghahramani, 2000). This feedback loop acts to allow refinement of the motor commands and movement execution with the intent of movement. As such, professional ballet dancers exhibit enhanced proprioceptive awareness to the upper limb (Ramsay and Riddoch, 2001), as well as the hip, knee and ankles, than non-dancers (Kiefer et al., 2013). Dancers also have a superior ability to integrate local proprioceptive information from multiple joints, than non-dancers, enabling them to form a more comprehensive understanding of the position of the limb in space (Jola et al., 2011). Furthermore, they are less reliant on visual information when integrating multisensory modalities about body position in space than non-dancers, enabling them the free flow of movement with exquisite finesse and without the restriction of maintaining visual fixation for feedback. This enhanced acuity enables them to perform highly complex movement sequences with short feedback latencies to the utmost precision.

### Peripersonal and Extrapersonal Space

The peripersonal space refers to the space immediately surrounding the body; the space in which we can grasp and manipulate objects in our environment. Conversely, the extrapersonal space is the space afar from us; the space we can never touch or reach into. Recent neuroimaging experiments have demonstrated that there are multiple representations of space constructed in the brain, which are rapidly being modified to accommodate our fast-changing environment (see di Pellegrino and Làdavas, 2015). These representations of space are centered on different body parts (i.e., hand-centered, head-centered, and trunk-centered) and are updated by incoming multisensory data. In non-human primates, the parietal, premotor, and prefrontal periarcuate cortical networks are thought to be integral to the peripersonal space representation network (Cléry et al., 2015). In the past, it was thought that the construction of these networks was based on actions in the near-body space, including hand and arm actions. More recently, work has shown that social and emotional contexts, as well as changes to the brain’s representation of the body, can also alter these peripersonal space representations (see Cléry et al., 2015 for a review).

There is experimental evidence suggesting that the representation of one’s body in the brain can be extended to include objects that are not a part of the body (Rademaker et al., 2014). The adaptability of the brain’s representation of the peripersonal space to accommodate for non-body objects and the space around those objects remains unclear (see Holmes, 2012). Yet for instrumentalists, their musical instruments might become “integrated” into their body representation and their peripersonal space may extend into the space surrounding their instrument. This is similar for athletes who use equipment, for example, – hockey players, tennis players, and basketballers. When coordinating actions with others, such as dancing with another, there is a thought that a representation of their actions and intentions is formed (Sebanz et al., 2006). This may also involve a “shared” representation of tasks, whereby there is a coordination of the partner’s actions with one’s own. The synchrony of a dance performance is highly dependent on precise anticipation of the partner’s actions, rather than just reacting to them. For performances of musical ensembles, this anticipation is of equal importance, allowing for precise timing, especially when the tempo is accelerated or slowed down. Non-verbal means of communication probably play a key role in updating these anticipations.

Dance is often performed with intimacy and passion – connecting two or more dancers in unison or as an outflow of personal emotion or the emotion of a character/role (for example, during professional dance). Given the intimacy, closeness and vulnerability often associated with dance, interactions within one’s peripersonal space are highly relevant. It is well-understood that we have a heightened protective system when threats enter within our peripersonal space (Sambo et al., 2012). It is possible that with heightened passion and vulnerability, our defensive system becomes downregulated. Even though one may not be likely to encounter threat during a professional dance performance – for recreational dancers at a large concert or festival flying arms or objects in a tight space (and therefore within one’s peripersonal space) are not uncommon scenarios. Indeed, in the case of potential threats both within (Sambo et al., 2012) and entering into (Wallwork et al., 2016) one’s near body space, we see an upregulation of defensive reactions (i.e., namely the “defensive peripersonal space”). This defensive peripersonal space is considered to have somewhat flexible boundaries and has been found to increase with increasing anxiety (Sambo and Iannetti, 2013) – a trait of many dancers anxious of onlookers and judgement. Interestingly, this defensive peripersonal space has the ability to adapt with changes in body position – it becomes heightened above the direction of gravitation pull (Bufacchi and Iannetti, 2016). That is, when standing upright, the defensive peripersonal space extends upward from the head in the direction against gravity, but when lying face-upward, it extends up and away from the face; suggesting an upregulation in the defensive system to accommodate for the potential for objects to fall toward the face along the gravitational pull. This is an important consideration for dancers and athletes, who regularly change body position and orientation often in fast changing environments, because it helps us to better understand how we interpret, perceive, and protect our intimate space. Given this boundary appears flexible, often adaptive to cognitive manipulations (Wallwork et al., 2016), it also encourages us to consider the possibility that this protective space is emotionally adaptable. This line of thinking is consistent with the above mentioned ability for emotion to alter the peripersonal space representation (Cléry et al., 2015).

### The Construct of the Body Matrix

The “body matrix” theory refers to the dynamic and widespread neuronal network that integrates multisensory data that include somatotopic body representations, spatial coordinates for the peripersonal space, as well as physiological regulation of bodily homeostatic systems (Moseley et al., 2012). Importantly, the body matrix is derived from a body-centered frame of reference, meaning that its representation is established and maintained regardless of the location and orientation of the limbs. For example, if the hands are crossed over, such that the left hand resides in the right side of space, there is likely to be a conflict in somatotopic and spatial representations. However, in this case, the left hand will still be allocated a right spatial reference. There are infinite number of inputs that can alter the “body matrix”, including sensory events (i.e., tactile, proprioceptive, auditory, visual, and vestibular) and cognitions (i.e., emotion and memory). In this paradigm, the body is perceived as a single entity and is represented as a multimodal construct with elements, which are more implicit (proprioception) or explicit (visual experience of body parts – usually the hands). The body matrix theory integrates a tight and functional connection between representation of body, space, cognition, and emotion; a conception that is fundamental in music and dance. The implications for performance include the ability to immediately transform into a character – whereby adopting the physical aspects of a role allows facilitation into emotional synchrony or vice-versa (Bellan et al., 2017). The neural correlates of the body matrix remain yet to be elucidated (Moseley et al., 2012); however, it is likely to involve widespread cortical networks. Tertiary neural areas, such as the IPS, are also likely to play an integrative role.

**Figure 2:**
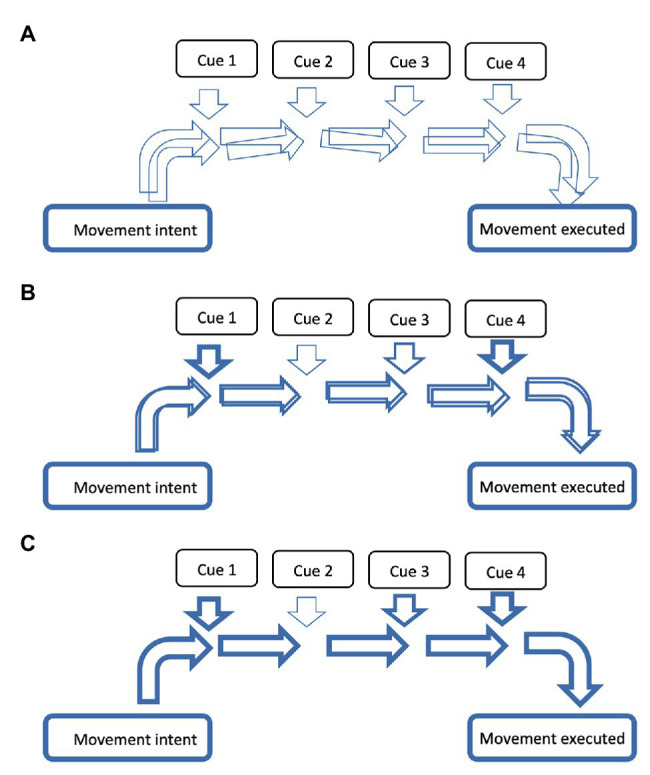
Stronger and more accessible neural networks are established with repetition. A simplistic schematic diagram demonstrating how the repetition of a neural network over time becomes stronger and more accessible. From movement intent (left side) to movement execution (right side), the more frequently a neural network is activated the stronger it becomes, as represented by increasing thickness of horizontal arrows from **(A)** through to **(C)**. In the first schematic **(A)** the arrows only have a thin outline (demonstrating it is a weak network) and several are overlapping (demonstrating its imprecision). With repetition of a given network, these arrows become thicker and more precise, as seen sequentially in **(B,C)**. Note the feeding of cues (vertical arrows) that might be associated with executing that movement – these cues could be multisensory cues, emotive cues, or cognitive cues. One cue might contribute more than others, as represented by thickness of arrows feeding from that cue. Note that the location of cue contribution is not a representation of time or order. If the movement intent is activated, but with different cues of different strengths feeding into the neural network with each separate execution, the neural activity will deviate to form a different neural network each time (with the consequence that the movement will be different each time it is performed). The end result is a movement execution that has a representational character, contributing to the “general idea” of a certain movement. When the neural network for a given dance sequence has been repeated several times, such that it is precise and easily activated (such as **C**), taking away specific cues, or reducing the input of specific cues, may not affect the overall activation of that network. However, it is likely that this will vary according to the dependency of that cue for the specific network and how strong the neural network is. For example, a dance sequence can be run with the dancers eyes closed (i.e., no visual feedback required), but is still dependent on the auditory cues.

The body matrix neural complex appears highly adaptable. During structural neural impairment, such as lesions to the inferior parietal lobe (see Gerstmann, 1930) elements that make up the body matrix, for example, the body position in space, become more dependent on other sensory inputs (such as the visual system) as a substitution for the impaired primary integration system. Furthermore, athletes, dancers, and instrumentalist may form strong associations between movements, sounds, emotions, and cognitive processes and as such, the supporting neural networks adapt by integrating and connecting the respective networks. For example, a certain tune might become associated with a specific emotion, such that whenever that tune is heard, that emotion is evoked. Importantly, the more frequently and precisely these “associative” networks are run, the stronger and more accessible they become (Wallwork et al., 2016). Frequent repetition of a dance routine or musical piece that becomes associated with other multisensory or cognitive/emotive cues, would result in a more reliable association between these components, which would be supported by a stronger and more precise neural network (see Figure 2 for a schematic diagram of this concept). Under the framework of the body matrix theory, body movement, music, and emotional expressiveness are intimately connected. These links may be related to the tone, tempo, and/or rhythm of the music, the personality of the role they are expressing, or the connectedness with the wider environment. The body matrix theory provides an accessible approach to understanding neural adaptability for optimizing performance.

### Inter‐ and Intra-Individual Synchronization

Synchronization with external pacemakers can be realized by acoustic or visual signals. Krause et al. (2010) were able to demonstrate that among musicians and non-musicians, drummers show the most accurate synchronization to auditory pacemakers. The authors observed that in general, musical expertise affected synchronization variability, such that the more experienced musician exhibited less variance in synchronization, indicative of improved timekeeping mechanisms. Similarly, dancers are more accurate at sensorimotor synchronization tasks than non-dancers (including tapping to audio, visual, and audio-visual stimuli; Jin et al., 2019). In an EEG study by Fujioka et al. (2015), distinguishable beta-band oscillations in bilateral auditory and sensorimotor cortices were found to be representing an isochronous auditory beat (either binary, march-like beat, or ternary waltz-beat). This was interpreted to mean that the brain was in some way expecting and anticipating the recurrence of the isochronous beat. But not only was the oscillatory activity modulated by the auditory beat, but also the extent of modulation that occurred was in fact reflective of whether the individual beat was perceived as accented or not. Moreover, in this study, the beat-related modulation of beta-band oscillations was also present when the participants were just imagining the beat. In a source analysis across the whole brain, the authors identified additional brain regions that were similarly affected in their beta-band oscillations by the perception of the beat. This included temporal, frontal, and parietal lobes, as well as the cerebellum. The authors concluded that these findings could reflect the translation of timing information to auditory-motor coordination. Vuust et al. (2014) applied the predictive coding theory to create a model for rhythm perception, a precondition for synchronizing processes in all musical contexts. The predictive coding theory was initially introduced by Friston (2005) to explain the hierarchical processing of sensory input in the brain. According to Friston, a sensory input is processed in order to infer its (most likely) cause, thus establishing the basis for perceptual learning. In general terms, this processing can be described as a mechanism to extract salient information from vast amounts of incoming signals in order to avoid processing of redundant information. According to Vuust et al. (2014), individual expectations are expressed in a predictive model, based on factors like experience and context. Perception occurs within this anticipatory framework and is used to update predictions when errors (mismatch between expectation/internal model and the actual input) are detected. Rhythmic expertise was found to have a negative correlation with the effort to maintain a metrical model when a counter-meter was introduced. This negative correlation was also true for the overall brain activity during the maintenance of the initial metrical model (Vuust et al., 2006, 2011), and was associated with bilateral activity in Brodmann areas 47 and 40. With the involvement of the non-dominant side, this activity was thought to be reflecting the processing of prediction error and representing “bistable” concepts (for stimuli with more than one possible interpretation, e.g., Rubin’s vase: Rubin, 1921) respectively. In summary, keeping pace with an external pacemaker is enabled by the maintenance of a predictive model. This predictive model can be seen as an internal simulation of events that are most likely to happen. The anticipatory framework that determines the quality of these predictions can be modulated by experience and training.

On a group level, human interactions based on rhythmic aspects of music can lead to neural entrainment (i.e., brainwave synchronization; Thaut et al., 1999). For example, some EEG studies have found synchronies of theta and delta brain waves when musicians played together (Lindenberger et al., 2009; Müller et al., 2013). Movement synchrony is developed from an early age (Leclère et al., 2014) and encompasses moving with a form of “togetherness” with a partner or cohesively within a group of people. “Group flow” occurs when performers simultaneously listen to themselves and each other while playing music (Sawyer, 2006). Phenomenologically, musicians frequently report on feeling a “sense of energy, rhythm or intuitive knowing” in a group when they act toward a common purpose (Cotter-Lockard, 2012). Reports on a strong sense of commitment are frequent in investigations on musical ensembles (Tovstiga et al., 2005; Blank and Davidson, 2007; Lim, 2014). When dancers perform in a group, a range of multisensory cues contribute to the quality of synchronous movement. For example, auditory coupling (having a musical beat to move to), tactile coupling (holding hands while dancing), and visual coupling (seeing fellow dancers move with you) contribute to the ability for a group to move in coordinated synchrony. Tactile coupling has been found to be the most powerful contributor for synchronous movement (Chauvigné et al., 2019). In some respects, this is not surprising given the known powerful effects of interpersonal touch (see Gallace and Spence, 2010); for example, its ability to influence social behavior, to create an interpersonal “bond,” and act as a powerful communicator of emotion. Synchronous movement can also be a form of social interaction. Oxytocin, a neurohormone, is thought to improve social interaction and coordination (IsHak et al., 2011), as well as the ability to empathize with others (Geng et al., 2018). In a study by Josef et al. (2019), oxytocin was investigated as a modulator of interpersonal synchrony during dance. Interestingly, those administered with intranasal oxytocin demonstrated heightened synchrony during dance, and this relationship was stronger for those with higher levels of trait empathy.

The temporoparietal junction has been proposed to be one of the key brain regions underlying interindividual synchronization processes (Heggli et al., 2019). Interestingly, the temporoparietal junction has also repeatedly been associated with a function called the “Theory of Mind” (Saxe and Kanwisher, 2003). In short, this is related to reasoning about the content of mental states. There is a vast amount of literature surrounding “Theory of Mind” and it is beyond the scope of the current review to delve into this here, but we raise it as we believe it does have some relevance to synchronizing processes in dance and music. “Theory of Mind” principles can be translated to dance synchronization due to its fundamental tenets of understanding, interpreting or predicting social interactions, beliefs, emotions, knowledge, and behaviors of others (Premack and Woodruff, 1978). Similarly, dance synchronization demands one to understand others’ intentions, behaviors, beliefs, desires, and other mental states, in real time, in order to maintain rhythmic harmony or choreographic sequence (see Gweon and Saxe, 2013 for a review in “Theory of Mind”). Lesions in the temporoparietal junction have been associated with out-of-body experiences, which are thought to be the result of a defective body schema, caused by disparate integration of perceptive input (Blanke et al., 2004). The temporoparietal junction also happens to be part of the vestibular cortex network (zu Eulenburg et al., 2012). The integration of vestibular information with visual, proprioceptive, and auditory information could be a good model for complex ongoing integrative processing in the brain. In addition, the integration of vestibular information can adapt fairly well to compensate for unilateral vestibular loss, suggesting a high flexibility of multimodal integration (Strupp et al., 1998; Helmchen et al., 2009). The vicinity of the processing of the body’s position with respect to gravitational influences and the construction of a “Theory of Mind” could be seen as quite literally standing in someone else’s shoes or looking through someone else’s eyes.

The complexity of the inter-relationship between cognition, bodily systems, and physical performance to promote synchronization processes, as outlined here, is remarkable and provides further evidence for cohesive and adaptable connection between body, space, cognition, and emotion (see above “The construct of the body matrix”). Future studies should investigate how and where emotional and social factors modulate the multisensory integration in synchronization processes in dance and music. Furthermore, studies should be directed at identifying the factors that can modify or foster different synchronization strategies or that promote high flexibility in switching strategies (Konvalinka et al., 2014). It would be interesting to empirically investigate synchronization patterns and strategies in more natural settings from music and dance performances and to correlate their occurrence with functional and structural brain characteristics.

## Specific Observations in Instrumentalists, Singers, and Dancers

### Music Production

When making music, bodily movement is much more than just transforming physical movements into sound waves – ancillary movements are related to individual interpretative choices and their communication (Davidson and Correia, 2002; MacLennan, 2015; Santos, 2019). Music is used to express ideas and emotions in manifold ways across different cultures. However, we here want to focus on the practical considerations of particular techniques of performance. When an instrument is being played, movements are not limited to hands and arms, as one might imagine (e.g., for the piano). On the contrary, the torso can be bent forward or leaned back, or the weight in the pelvis may be shifted from one side to the other. These movements are not necessary in terms of sound production, but they are introduced to enhance the performance. At first, this might seem counterintuitive, because they add complexity to already complex movements. However, these movements can be integrated by means of rhythm/beat or emotional content expressed in the music. When looking at the biomechanical analyses by Gärtner (2013), who investigated the relationship between sound and force production in piano playing, the key point seems to be to visualize (or rather, auralize) the goal (i.e., the desired sound), rather than deliberately choosing a certain movement, which is thought to produce the desired sound. Most likely, this connection is best made when audio-motor associations have already been formed. In the production of music, intentionally produced auditory output is monitored to guide sound-related actions. The different processing of intentional compared to incidental sound production has been investigated by manipulating the visual and auditory feedback of tap dancing and hurdling (Heins et al., 2020). Attenuation of self-produced sound input was stronger during intentional sound production (tap dancing) than during incidental sound production (hurdling). The authors argue that the auditory action consequences are applied in a predictive model using already existing associations between sound and movement. Here, a low prediction error is thought to cause the attenuation of actual sensory input that matches the predictions (see “music perception” above). In an exploratory approach, Cotter-Lockard (2012) evaluated the specific musical rehearsal techniques that were used in a chamber music coaching process. She found that many of the techniques were designed to evoke an embodied experience, for example, feeling and expressing the meter of the music by actually moving the body, thus increasing the bodily (somatic) awareness. Other musical skill development techniques were aimed at linking awareness of the different senses, for example, visual and physical awareness. This could either improve the scope and switching of attentional focus or amplify non-verbal means of communication and expression. Another key technique included switching roles of leader and follower and in general encouraging a change of perspective. The overall conception of integrating all the modalities is striking. To teach conceptual ideas, different kinds of metaphors were used (physical and verbal). In particular, the picture of moving a ball of energy across the room by playing music is clearly demanding in terms of imaginative abilities. Moreover, this technique should enable the performers to agree on a common goal of abstract nature and change it over the course of a performance merely through non-verbal means of communication (e.g., deciding on where and when to move the energy next). Essentially, when performing music in a group, body, and eye gestures are used as a means of communication on interpretative questions that are continually dealt with during both rehearsals and the actual performance on stage (Davidson and Good, 2002). The total absence of interindividual touch in instrumental group performances may add further to this strong communicational need.

Evidently, despite the intricate patterns of movement associated with music production, overall processing demands are kept at a manageable level. Prediction error is here introduced as a relevant gatekeeper for division of attentional resources. Using abstract ideas like metaphors to subsume all the performance elements under one common cognitive cue is a strategy that is actively applied. Both mechanisms could reduce information complexity to manageable chunks of multimodal information. This would explain (a) why experts frequently show less brain activity than amateurs and (b) why it seems to the audience that they should have immense working memory capacity to be able to perform at such a high level of complexity. In a “choreographic performance approach,” where movement integrates both ergonomics and individual interpretation, the IPS might serve as an integrative hub, providing one of the preconditions: multimodal information has to be integrated efficiently to keep overall complexity manageable and to enable complex performance.

### Learning to Dance: Rehearsed vs. Freelance Dance

Anyone can learn to dance. Whether or not you are any “good” at it is a personal and subjective opinion. Much like art, dance can come in almost any shape or form and a lot of modern dance is interpretive. Dance, being a performing art that is often, but not always, performed to music, can be learned at an early age, often through the observation and mimicking of other’s actions, namely “imitation learning” (Rizzolatti and Craighero, 2004). When observing an action of others, it is believed that neurons that represent that action become activated in the observer’s premotor cortex and a motor representation of that action is formed (Rizzolatti et al., 2001). In this way, the visual information becomes interpreted into knowledge in the way of a movement representation. It is thought that this is done through mirror neurons, a class of visuomotor neuron in the parietal and premotor cortex (Rizzolatti et al., 2001). Observing and understanding an action or movement activates this mirror neuron system. Brain imaging studies on humans suggest that the mirror neuron system includes a dynamic and complex network including the occipital, temporal, and parietal visual areas (bilaterally) as well as cortical motor regions – specifically the rostral part of the inferior parietal lobule, the lower part of the precentral gyrus, and the posterior part of the inferior frontal gyrus (Buccino et al., 2001). Interestingly, the motor cortex becomes activated even in the absence of any overt movement from the observer (the “motor resonance system”; see Rizzolatti et al., 2001 for a review).

When learning and then rehearsing a dance routine, there appears to be a clear transition in neuronal activity over time. Bar and DeSouza (2016) conducted a longitudinal fMRI study, where professional dancers learned and then rehearsed a new dance routine over a 34-week period. At certain time points over the 34-weeks, the dancers were asked to visualize the dance routine to music while undergoing fMRI scanning. Interestingly, the authors found that there was increasing activity in the SMA and auditory cortical regions during the first 7 weeks of rehearsal, which then decreased over time with further practice (Bar and DeSouza, 2016). In line with this, there was an increase in activity in some subcortical regions, such as the basal ganglia, which the authors point out has been recognized for its involvement in habit formation and memory (Packard and Knowlton, 2002). That is, it is quite possible that the learned choreographed movement sequences may be maintained by subcortical structures, at which time the cortical regions then become less involved. Furthermore, it is speculated that the decline in cortical activity after the initial learning phase may be due to a reduction in activity in those regions; however, it is also raised that this decline may also be evidence of enhanced neuronal efficiency. The finding of Bar and DeSouza (2016) is perfectly in line with a TMS and fMRI study of our group were we reported that training effects can be modulated by cortical theta burst stimulation during the first days of daily training but not after more than a week of daily practice (Platz et al., 2018). fMRI confirmed that cortical processing during performance of left hand motor tasks decreased over time in the training group whereas basal ganglia activation (especially in the putamen) increased (Walz et al., 2015).

Dance can be impromptu, whereby “dancing” is done in an unstructured, free, and impulsive manner with one’s own creativity, emotion, and imagination for that time and place. When dancing with someone or several people in an impromptu manner, each dancer feeds off the actions of others to form (or attempt to form) a coordinated movement pattern. The “Theory of Mind” principles are again relevant here, as dancers are likely to acquire information to predict behavioral responses from others thoughts, beliefs, and feelings of others through social interactions (Byom and Mutlu, 2013). There are likely many complex processes associated with coordinating impromptu dance with others, as well as the integration to musical beats that promote temporal synchronization between dancers, however, the ability to perceive various social cues and infer behavior from these cues seems worthy of further investigations in this context. This is consistent with the idea, as raised earlier, that there needs to be a mutual understanding of the intentions and goals of others (Sebanz et al., 2006).

**Figure 3:**
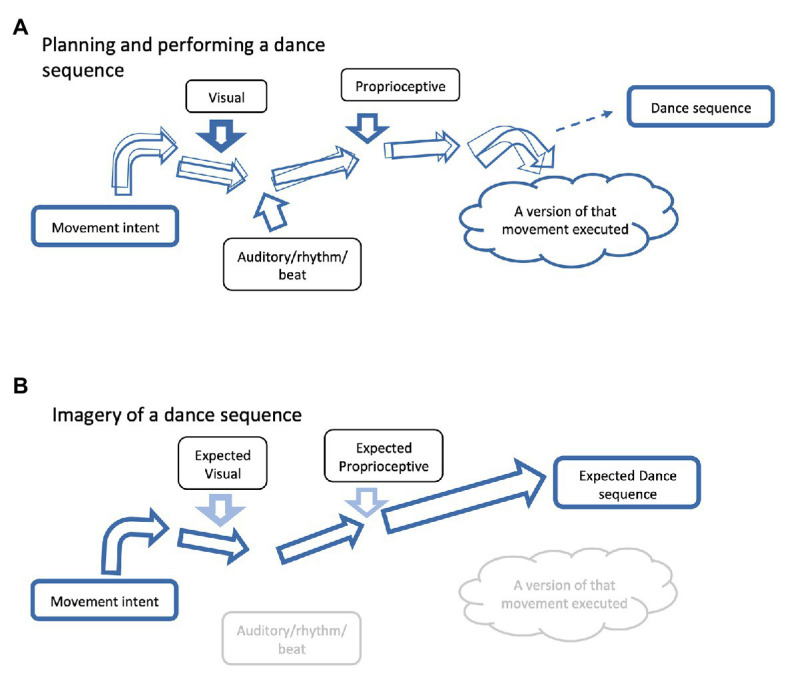
Imagery can be used to refine neural networks. A schematic diagram representing how imagery can assist in refining neural networks for dance and coordinated movement. Note that roughly horizontal arrows represent the neural network from the “Movement intent” to the “Intended movement.” The thickness of the horizontal arrows represent the strength of the neural network (i.e., the thicker the arrow the stronger the network) and the overlapping arrows represent the precision of the neural network (i.e., the more congruent the arrows, the greater the precision). **(A)** A schematic diagram of a neural network for a given movement. A movement can be influenced by a number of different cues (as represented within the boxes) of differing strengths (as represented by the thickness of the roughly vertical arrows). With practice, the network will become stronger and more precise, as long as the cues remain relatively consistent. As the cues become more refined, so does the network. As the network becomes stronger, external cues/perturbations are less likely to influence the neural network and therefore the outcome. **(B)** Imagery of that same movement can be done in a more controlled environment. Contextual cues can be reduced to allow greater focus on the movement at hand. Once the neural network for that movement is stronger and more precise, other contextual factors can be added in the imagery process. Note that visual and proprioceptive cues are anticipated cues, as true feedback is not possible in the imagery environment. Also, note that the movement is not executed, rather it is the expected movement. Executing the actual movement is necessary for refinement in a real-life context, but imagery and visualization can help to reinforce the neural network without extraneous distractions.

Repeated rehearsal of choreographed dance routines likely engages different processes to impromptu dance. Very specific movements through time and space are brought together to form dance routines that are practiced time and time again until they are finessed to the point that they become automatic. Professional dancers use several visualization or imagery strategies to enhance their performance. This might include the use of mirrors to enhance their real-time visual feedback, watching others or themselves through pre-recorded material (i.e., action observation utilizing the mirror neuron system) or through motor imagery practice. The latter involves imagining oneself moving through the sequences (see Gatti et al., 2013 for a mini review on action observation and motor imagery), often in synchrony to the relevant music (which is often also in their imagination), while also focusing on the “feeling,” “mood” or any other contextual factors associated with that performance. It is thought that this can help to reinforce these neural associative networks, such that they become more automated, so that focus can be oriented elsewhere for further refining of performance (see Figure 3 for a conceptual schematic diagram on how imagery training can assist in refining neural networks to enhance performance). Furthermore, some dancers will dance with their eyes closed, a method that enhances one’s focus on the proprioceptive cues and integration of body awareness. Such strategies of motor learning have also shown some promise in neurorehabilitation for conditions such as Parkinson’s disease (Caligiore et al., 2017), post stroke (Emerson et al., 2018), and are gaining momentum in people with persistent pain (Thieme et al., 2016; Wallwork et al., 2016; Suso-Martí et al., 2020) to reduce movement-related disability.

Although the quality of the motor execution is clearly important for professional dancers and musicians, it must be recognized that psychological factors, including focus and psychological well-being, play a vital role in professional dance and musical performance. More specifically, this might include motivation, perfectionism, injury and pain, personality types, social and professional relationships, anxiety and stress, memory, self-confidence, and self-esteem (Nordin-Bates, 2012). Therefore, although not the focus of the current review, it is important to consider and acknowledge the wider contributions that psychological variables can have on performance.

## Bringing Together These Single Observations to a Basis of Knowledge

The different aspects of coordinated complex movements in dance and music performance outlined above illustrate how the brain is able to integrate multiple streams of sensory and spatial information to encode and retrieve very complex yet highly robust movement sequences. In this section, we will elaborate on how the individual aspects of spatiotemporal movement coordination through multimodal feedback integration can be fed into a common framework of information processing located in frontoparietal networks. Further, we will discuss techniques that could be useful to enhance this network in order to improve the teaching and learning of complex multimodal tasks.

### The Anatomy of Motor Control: Retrieval and Adaptation of Motor Programs

As outlined early on, our brains are wired to integrate multimodal information effectively from a young age. Even incompatibilities as in the case of temporal resolution discrepancies between aural and visual information can be overcome. Basic motor learning processes have been studied extensively and can be explained by basic theories like Hebbian learning. On the contrary, much less is known about complex motor performance; how it is learned and taught and how the brain controls and modifies it. From the philosophical perspective, Dreyfus (2002) explains that the beginner applies a set of explicit rules to master a novel task. With advanced learning stages, the performer gains experience by collecting exemplary situations in which the rules can be applied. With growing expertise, the learner will gain independency from explicit rules and start to apply implicit rules that have been subconsciously formed by experience. Applying Dreyfus’ rationale about expertise in motor learning research, we find that our repeated experiences lead to the creation and updating of likelihood mappings of associations between actions and the respective associated feedback. These mappings have been termed “predictive error,” as described above.

Turning toward neuroanatomy, we are now in need of a sufficient anatomic model that comprises the retrieval and manipulation of complex motor programs with their corresponding mappings of feedback associations. While retrieval of motor programs and temporal dynamics of preparatory brain activity in general have been studied quite extensively, the actual content of the preparatory processes has been somewhat neglected in research of sensorimotor learning. Following the implication that deeper knowledge of the content of motor preparation could also help in explaining effects of imagery-based motor learning, the details of preparatory processes are of the highest interest for research of complex sensorimotor learning. Applying current anatomical and functional knowledge of the human brain and associated behavioral consequences, in the following, we will synthesize a model for complex motor learning processes based on the retrieval and modulation of motor programs.

Network analyses have shown that a frontoparietal network acts as a major hub in the human brain. Importantly, this network dominates most other networks that have been described and is thought to be capable of modulating their activity (Cole et al., 2013; Marek and Dosenbach, 2018). The frontal elements of this network exercise cognitive control over subordinate processes by controlling the attentional resources. In monitoring ongoing actions, sustained attention has been associated with activity in the dorsolateral prefrontal cortex (PFC; Schnell et al., 2007), with BA 9 (as well as the parietal cortex) being additionally involved in probabilistic assessments (Huettel et al., 2005). On the contrary, activity in the ventrolateral PFC has been shown to correspond with the detection of visuomotor incongruencies (Schnell et al., 2007). In a study of visual spatial attention, for example, the frontal eye-field is expected to exert control over the IPS, for which a functional connection has been demonstrated (Szczepanski et al., 2013). The IPS is known to be engaged in multisensory integration for movement planning as well as for spatial orienting of movement execution in relation to objects. For example, when planning an eye-movement in order to detect a stimulus in a certain location (e.g., a flying bird), the motor control system would refer to a set of spatial templates from the IPS to estimate the most likely location of the stimulus (e.g., the sky). The IPS is also known to maintain attentional sets, like an object-specific or spatially oriented attentional focus (Scolari et al., 2015). Similarly, we anticipate that the differential weighing of a set of feedback cues, as it is established during repetitive practice (for the example of dancing see Figure 3), should be stored in the IPS. These presets could then be used to create a feedforward prediction of expected feedback for motor planning. Based on previous experience in the task at hand, a template of expected feedback is likely to be retrieved during the preparatory phase of movement planning in both motor imagery and physical performance scenarios. Motor expertise would determine the number and precision (expressed as a predictive error) of available templates. Experience in imagery techniques should determine the number of modalities that can be evoked and monitored successfully during a simulated action.

From the study of groove, we know that frontoparietal networks also play an important role in connecting music perception and movement (Matthews et al., 2020). While prefrontal activity is associated with the accuracy of predictions of sensory events (Bengtsson et al., 2009), parietal areas integrate sensory input from different sources to provide a framework of actually perceived feedback combinations. Based on this framework, likelihood estimates about future events can be calculated. It is also important to mention the role of subcortical structures, such as the basal ganglia, which likely play a role in the automatization of complex learned motor patterns (Bar and DeSouza, 2016). Furthermore, the neural connection to the striatum in the basal ganglia links the expectations to the realization of movement plans. Specifically, the ventral striatum had been associated with an overlap of expectations with one’s own performance (Filimon et al., 2020).

### Skirting the Limitations of Attention and Working Memory in Expert Performance

Similar to computers, working memory is a limiting factor when it comes to complex operations in the human brain. Forming multimodal sensory associations during learning of complex movements may be a mechanism to minimize the processing demands during retrieval of a motor engram. Experiencing a highly reproducible relationship between an action and the resulting feedback (e.g., the relation between finger position on the instrument and resulting pitch of the produced output) can be expected to lead to the establishment of action-feedback associations with very low predictive error. For example, if a certain key is pressed repeatedly on the piano, the resulting pitch will remain the same. Environmental conditions could be altered (i.e., the room in which the piano stands, the lighting, and the temperature of the air) without affecting the relation between key and pitch. The movement pattern that connects the individual keypresses in a both ergonomic and economic way is then implemented in this steady position, with the thumbs of both hands anchored on the “C4” (middle C) in the case of the piano, or in the first position on stringed instruments, where the left hand is placed on the most distant part of the fingerboard. Concerning the motor program, the lower number of degrees of freedom in the movement of pressing down the keys on a keyboard compared to the complex bimanual interaction required to produce a tone on a stringed instrument seems advantageous in the early establishment of motor-feedback associations. Koelsch et al. (2019) postulate that a low prediction error in musical contexts (additionally modulated by culturally primed expectations; see *Music perception* above) should lead to increased gain for the processing of any mismatching information. This attentional gain allows for sustained attention toward error detection. Importantly, the use of predictive (feedforward) projections may lead to reduced processing on higher cognitive levels, as suggested by Gill et al. (2008) in a study on auditory processing in the Zebra Finch: when highly reliable predictions are available, only discrepancies in actual feedback signals are processed, while little attention is lost on confirming all the correct predictions. Hypothesizing that attentional resources can be effectively managed when sensory information has a high reproducibility (i.e., low prediction error), we may assume that the integration of multiple feedback modalities in motor planning should at some stage of learning result in a reduction of prediction error compared to motor learning with unimodal feedback. For example, a movement that evokes the expected somatosensory sensation and visual feedback of the finger pressing down on the string can be corrected more efficiently if an auditory mismatch is detected compared to feedback of a singular modality, which may lack in precision. The question that arises from the didactic standpoint here is whether it is possible to actively foster error detection techniques like “active listening” or whether improved error detection arises as a mere byproduct from motor expertise itself, as it is achieved through repetitive training. We hypothesize here that deliberate change of attentional focus toward different feedback modalities (i.e., sound, proprioception, and vision; see Mantel, 2013 for instrumental teaching approach) could be an effective method to improve error detection.

Using motor imagery of single modalities could further enhance the ability to switch the attentional focus to individual feedback modalities during actual performance. Athletes, dancers and musicians already apply this technique of rehearsing mentally without moving. It is believed that this kind of “silent” rehearsal stabilizes the representation of movements particularly with regard to the order of movement sequences (e.g., as in high diving; Reed, 2002).

**Figure 4:**
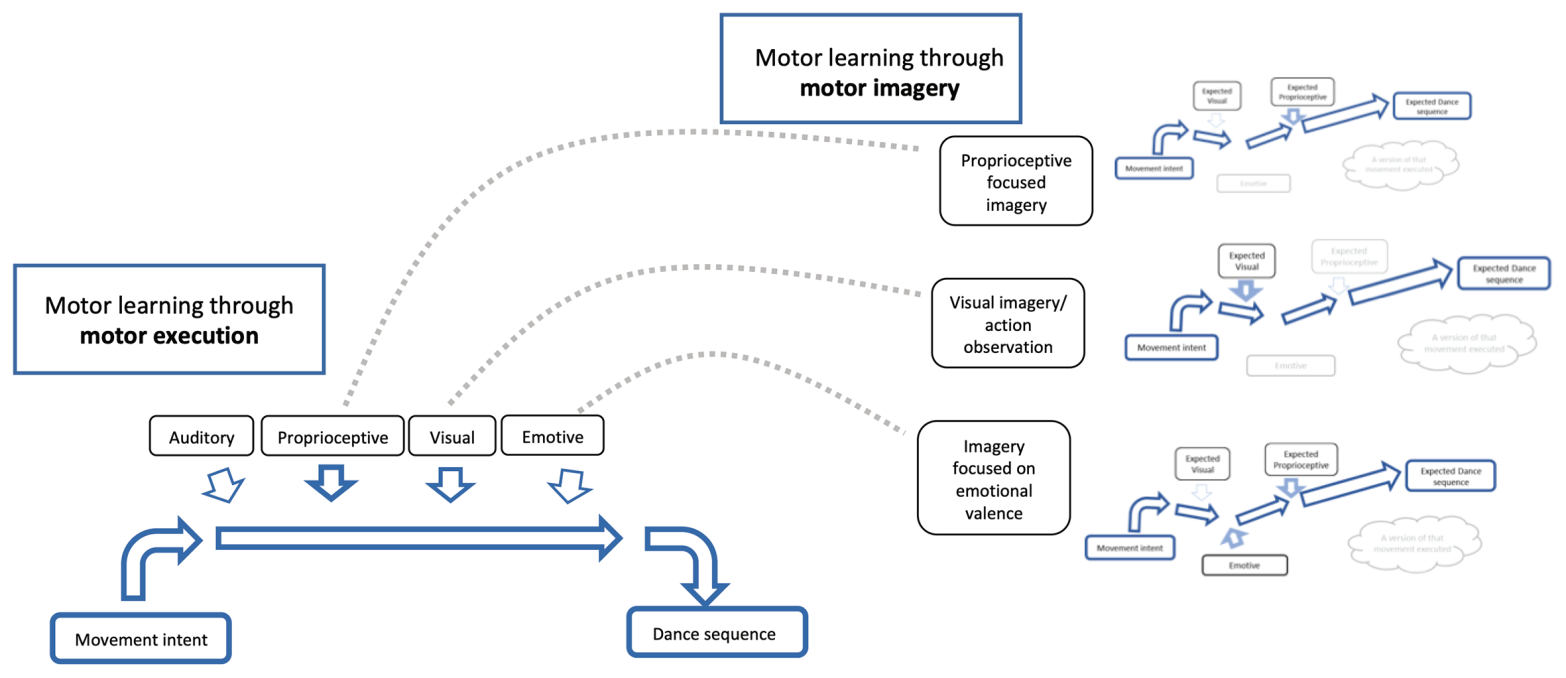
Imagery training can be cue specific as a strategy to enhance performance. Schematic diagram illustrating neural networks for motor learning through motor execution (left side) and motor learning through motor imagery (right side) and how motor imagery can assist in refining a motor output, such as dance. If a dance sequence is practiced over and over, the neural network for that dance sequence will become stronger and more precise (see Figure 2, above). If the neural network for that given dance sequence is rehearsed using motor imagery, the cues of focus (i.e., proprioception and visual) will also become stronger, as will the links between the cues and the dance sequence (see Figure 3, above). Imagery training can be cue specific, for example, focus can be directed to the proprioceptive representation of that dance sequence (i.e., focus on what the movement “feels like”; see top right) or directed toward the visual representation of the dance sequence (i.e., what the movement “looks like” – could also include action observation; see middle right), or directed toward the emotive valence (i.e., the emotion associated with that dance sequence; see bottom right) and so on. There are infinite number of cues of focus for imagery that will help with the overall motor learning and execution of that dance sequence. Imagery training (right side) assists in strengthening and refining the neural network, which translates into the real motor execution (left side; see gray dotted lines moving between imagery and motor execution for a given cue). With practice, imagery can be performed with a focus on two or more cues, as the connections and associations of the neural networks get stronger and more precise. Note that motor learning through motor execution needs to be maintained to strengthen the relationship between imagery and execution neural networks.

Compared to actual motor execution training, state estimation in motor imagery may be less precise because of the lacking feedback modalities (spatial attributes, proprioception, and velocity; Gentili and Papaxanthis, 2015). Despite its comparably small direct effects on motor performance itself, mental practice may be an effective tool to develop multimodal feedback pattern registration in motor learning. By adding feedback modalities in a controlled and stepwise manner during imagery practice, formation and retrieval of elaborate feedback patterns could be enhanced. In experts with established extensive cross-modal associations, the retrieval of such a multimodal feedback template could be initiated using a single modality cue (see Figure 4). In the advanced learning stages, such a singular “super cue” could even be a simulated feedback cue from memory (i.e., using imagery). Our proposed concept of a super cue is comparable to the function of a keyword in semantic memory retrieval. In the case of semantics, a keyword evokes memories of associated words. For our concept of cross-modal associations in movement programs, the imagery of movement-associated auditory feedback could serve as a “super cue” to recall both the motor program and any associated feedback from multiple modalities (e.g., visual and kinesthetic). The retrieval by a single modality cue is opposed to the initially introduced retrieval of a motor program by synchronously perceived feedback from multiple modalities. Testing these ideas on the use of motor imagery in stepwise formation of multimodal sensory-motor associations could be a stepping-stone toward the study of complex motor learning. Applying this structured technique in multimodal motor learning may add to the robustness of a motor program, allowing for stable performance levels in fast-changing environments like a dance performance in a group or performing in a symphonic orchestra.

### Multimodal Processing as a Precondition for Expert Performances

Multimodal processing is a key feature of the two models introduced above: the body matrix is a body-centered framework integrating body, space, cognition, and emotion, and the Theory of Mind, on the other hand, serves as a basis for inter-individual synchronization and communication by extrapolating anticipations beyond the self, toward other individuals. Typically, two different goals are merged in the motor performance related to the arts of dance and music: movement is necessary to produce sounds or move through space on one hand (e.g., sounds on the instrument or to move across the stage in dance performances), but on the other hand, higher order cognitive and emotional ideas are expressed by these movements too. They are conveyed to both the audience and co-performers. The latter helps in synchronizing, while the former enhances the communication of ideas that are encoded in the music or dance choreography itself. One practical example for the piano is the assignment to imagine the sound of the down-pressed key to expand and crescend. From a physical standpoint, this is impossible, because the string inside the piano is hit on one instance only. While the movement of down-pressing is sufficient to produce the sound in an acoustic sense, the additional imagination of the temporal dimension of the sound may help to maintain the meter and to connect individual keypresses to create a melody. Furthermore, anticipation of future acoustic events through visible movement (like arching over the piano or leaning back) may help to overcome the instantaneous nature of music and prepare the audience for the upcoming phrase to enhance their individual experience. The communication with the audience could here result in enriched expectations about the music, strengthening its effect.

In relation to synchronization, two types of information are processed to anticipate behavior in a group performance: cognitive knowledge about the sequence of events (as it is incorporated in either a musical syntax or a choreographed routine) is combined with empathetic anticipation of the individual interpretation (variation) that will deliberately be imposed during the performance of the predefined movement sequence. While the predictive coding approach accounts for the way these anticipations may be generated in the case of sensory events, we can at this point only speculate on how and where the empathetic estimation of future events may be represented in the human brain. This would be an interesting idea to develop further in line with the body matrix and the Theory of Mind.

## Conclusion

We have outlined the fundamental principles of sensorimotor integration processes in the human brain based on current research literature. Using the examples of dance and music production, we modeled motor learning of complex movement performance and compared motor execution with motor imagery processes. By discussing a common framework of motor planning in frontoparietal networks, integrating both physical and imagery performance, we developed the base for investigating different approaches of teaching and performance in the fields of music and dance. We hereby provide guiding hypotheses for future research. Based on our model (see Figure 4), we hypothesize that retrieval and modulation of motor engrams with corresponding anticipatory feedback templates may be possible by unimodal cues like an auditory cue. A “super cue” from higher order cognitive brain regions (e.g., a simulated cue from memory content) may have the potential to retrieve and modulate motor engrams and corresponding feedback anticipations. This thought encourages further pursuit into research of teaching and performance techniques aimed at establishing multimodal associative movement engrams, both from a practical and theoretical viewpoint.

## Data Availability Statement

The original contributions presented in the study are included in the article/supplementary material, further inquiries can be directed to the corresponding author.

## Author Contributions

SW and AL contributed equally in writing of the manuscript. ML contributed in writing and organization of the manuscript. All authors contributed to the article and approved the submitted version.

### Conflict of Interest

The authors declare that the research was conducted in the absence of any commercial or financial relationships that could be construed as a potential conflict of interest.
